# Uncovering incontinentia pigmenti: From DNA sequence to pathophysiology

**DOI:** 10.3389/fped.2022.900606

**Published:** 2022-09-06

**Authors:** Kang Nien How, Hazel Jing Yi Leong, Zacharias Aloysius Dwi Pramono, Kin Fon Leong, Zee Wei Lai, Wei Hsum Yap

**Affiliations:** ^1^Dermatology Unit, Faculty of Medicine and Health Sciences, Universiti Putra Malaysia, Serdang, Malaysia; ^2^Dermatology Unit, Hospital Pengajar Universiti Putra Malaysia, Serdang, Malaysia; ^3^School of Biosciences, Taylor's University, Subang Jaya, Malaysia; ^4^Institute of Molecular and Cell Biology, A^*^STAR, Proteos, Singapore, Singapore; ^5^Paediatric Dermatology Unit, Department of Paediatrics, Women and Children Hospital Kuala Lumpur, Kuala Lumpur, Malaysia; ^6^Centre for Drug Discovery and Molecular Pharmacology, Faculty of Health and Medical Sciences, Taylor's University, Subang Jaya, Malaysia

**Keywords:** incontinentia pigmenti, *IKBKG/NEMO*, NFκB pathway, pathophysiology, molecular diagnosis, genotype-phenotype

## Abstract

Incontinentia pigmenti (IP) is an X-linked dominant genodermatosis. The disease is known to be caused by recurrent deletion of exons 4–10 of the Inhibitor Of Nuclear Factor Kappa B Kinase Regulatory Subunit Gamma (*IKBKG)* gene located at the Xq28 chromosomal region, which encodes for NEMO/IKKgamma, a regulatory protein involved in the nuclear factor kappa B (NF-κB) signaling pathway. NF-κB plays a prominent role in the modulation of cellular proliferation, apoptosis, and inflammation. *IKBKG* mutation that results in a loss-of-function or dysregulated NF-κB pathway contributes to the pathophysiology of IP. Aside from typical skin characteristics such as blistering rash and wart-like skin growth presented in IP patients, other clinical manifestations like central nervous system (CNS) and ocular anomalies have also been detected. To date, the clinical genotype-phenotype correlation remains unclear due to its highly variable phenotypic expressivity. Thus, genetic findings remain an essential tool in diagnosing IP, and understanding its genetic profile allows a greater possibility for personalized treatment. IP is slowly and gradually gaining attention in research, but there is much that remains to be understood. This review highlights the progress that has been made in IP including the different types of mutations detected in various populations, current diagnostic strategies, *IKBKG* pathophysiology, genotype-phenotype correlation, and treatment strategies, which provide insights into understanding this rare mendelian disorder.

## Introduction

Incontinentia Pigmenti (IP; OMIM 308300), an ectodermal dysplastic disorder, is a rare type of X-linked dominant genetic disease. It is caused by mutation of the *IKBKG* gene, which is located at Xq28. It encodes a vital component of the transcription of the nuclear factor kappa B (NF-κB) signaling pathway (1, 2). IP occurs in approximately 1:40,000 to 1:50,000 births (3, 4). The disease has a prevalence of approximately 0.7/100,000 with female patients being the affected population (5). Two major allelic mutations have been described, namely amorphic allele and hypomorphic allele. In amorphic allele, female survival is attributed to selective skewed X chromosome inactivation. This type of mutation is generally lethal in males, except for cases with XXY chromosome disorder or individuals with somatic mosaicism. On the other hand, the hypomorphic allele leads to mild IP in females, and affected males suffer from ectodermal dysplasia with immune deficiency (EDA-ID) (6). According to the biobank for IP (IPGB, https://www.igb.cnr.it/ipgb/) more than 75% of female and all male IP cases are sporadic (7).

IP can be clinically diagnosed based on the updated Landy and Donnai diagnostic criteria. This involved characteristic cutaneous manifestation, and abnormalities found on hair, nail, central nervous system, eye, orodentofacial, nipple, and breast. These criteria also take into consideration previous male miscarriages and family history of IP (8). Cutaneous manifestation occurs within the first few weeks of life. The skin lesions evolve through four stages, which begin with vesiculobullous eruption (Stage I), followed by verrucous stage (Stage II), hyperpigmented stage (Stage III), and atrophic, hypopigmented stage (Stage IV) (5, 9, 10). Not all stages occur and overlapping clinical manifestation is not uncommon. Eye anomalies are found in 35% of the patients. Retinal anomalies are by far the most common and include retinal vascular anomalies, retinal detachment, and retinal pigment epithelium anomalies. Areas of ischemia may induce neovascularization, which lead to gross intraocular scarring with severe visual loss (11). CNS anomalies can occur in up to 30% of IP patients. They commonly start in the early infantile period and adult-onset neurological symptoms are unlikely related to IP. Convulsive disorders are found to be the most common, followed by paralytic disorders, motor impairment, and intellectual disability. Other manifestations include odentofacial, breast, hair, and nail presentations. On rare occasions, skeletal, cardiac, and other organs may be also affected (12–14).

This review outlines current understanding of molecular diagnosis, pathophysiology, and the genotype-phenotype correlation.

## Current technologies for the diagnosis of incontinentia pigmenti

Analytical approaches for the molecular diagnosis of IP should be approached by considering the index case's gender. This is because in an IP female the variant is in a constitutively heterozygous state. This indicates that it can be found in all cells in the body. However, if the postzygotic mutation occurs in a male population, embryonic mosaicism allows two groups of genetically distinct populations to coexist in the same individual (6). In addition, the cell expressing the *IKBKG/*NEMO variant may gradually be eliminated and finally cleared, making the diagnosis in male patients extremely difficult. Simple PCR to detect the genomic deletion of exon 4–10 remains the recommended technique for *IKBKG* variant screening as the recurrent deletion account for 79% of female IP case (15) or 70% of total IP cases (7). If the exon 4–10 deletion is not detected, Sanger sequencing can be used to screen for point mutation and indel along the *IKBKG* coding region and intron-exon junctions will improve diagnostic sensitivity by 9%. On top of those above, qPCR can be employed to detect larger arrangements other than the classical exon 4–10 deletion that account for about 4% of IP cases (7). Despite next generation sequencing being more widely available and cheaper, it has been deemed unusable for IP diagnosis due to the presence of the pseudogene, *IKBKGP1*. Both *IKBKG* and *IKBKGP1* are located in the Xq28 region within and share 99% of their identity (16). The presence of this pseudogene makes the traditional capture probe data analysis difficult, as it reduces the read depth, decreases the mapping quality, and contributes to a poor alignment read, resulting in false-positive results (16, 17). However, a bioinformatics tweak masking the *IKBKGP* in the Next Generation Sequencing (NGS)/Whole Exome Sequencing (WES) pipeline analysis harnesses the technology, acting as a powerful tool in detecting mutations in *IKBKG* (18). With such innovations, NGS/WES undoubtfully accelerates the *IKBKG* mutational screening as an alternative to or in addition to the traditional Sanger (19). Low level mosaicism that happens in male patients, may escape molecular investigation if methodology in relation to female patients is used. Rather than having the genomic DNA extracted from peripheral blood, testing should be done using the tissue of choice from the suspected phenotype (i.e., skin) and analysis of multiple tissues, namely blood, fresh skin, saliva, and sperm samples to detect low-level mosaicism (7, 8, 20). Thus, the latter is more expensive and requires more specific competencies and infrastructure (19, 21).

## Incontinentia pigmenti: Genetics and pathophysiology

### Genetic variants of incontinentia pigmenti in various populations

The most common genetic mutation in IP is an approximately 11.7-kb deletion in the *IKBKG* gene that removes exons 4 through 10. This mutation accounts for 70–80% of patients with IP worldwide (22–24). This is found in European (25–27), Chinese (24, 28), Japanese (29–31), Korean (32, 33), and Indian (34) populations (Supplementary Table 1). Apart from the 11.7-kb deletion, IP can also arise due to other types of mutations along the *IKBKG* genes that include single nucleotide substitution, point mutation, and small insertion/deletion (indel). A point mutation can be a non-sense mutation that leads to premature protein translation termination or a missense mutation that leads to amino acid change. Small indel may lead to frame-shift or in-frame amino acid deletion. Both point mutation, as well as indel, may also cause aberrant splicing of the *IKBKG* mRNA. These mutations can result in the absence of or defective *IKBKG* protein, which yields a phenotype of IP (24, 28–31). Other than mutations involving exons, a single nucleotide polymorphism involving intron 8 was also reported by Chinese populations (28). Though less commonly reported, this polymorphism was also reported among Caucasian populations (35). While most reports on IP cases came from western population cohorts and certain East Asian regions, IP cases have also been observed in other populations such as African (36, 37), Indian (34, 38, 39), Malaysian (40), and Brazilian (41).

### *IKBKG* pathophysiology in incontinentia pigmenti

The IκB kinase (IKK) protein complex comprises the catalytic subunits IKKα and IKKβ, and IKKγ (NEMO) (42). The *IKBKG* gene is responsible for encoding for IKKγ (NEMO), which is responsible as the regulatory subunit of the inhibitor kappaB (IκB) kinase (IKK) complex essential for NF-κB pathway activation required in many elementary physiological functions (43). IκB protein phosphorylation, ubiquitination, and degradation upon the activation of the IKK complex results in the removal of the inhibitor that activates the NF-κB complex (44). The absence of IκB allows NF-κB to translocate into the nucleus, where the transcription of targeted genes can occur. Activated NF-κB has been reported to execute immune and inflammatory responses and is involved in the protection against apoptosis induced by signaling proteins (30, 42, 45–47). Thus, a lost-of-function or absence of the *IKBKG* gene contributes to the dysfunction of IKK and consequent termination of NF-κB activity. Without NF-κB, IP cells are highly sensitive to pro-apoptotic signal (43, 48–51).

**Figure 1:**
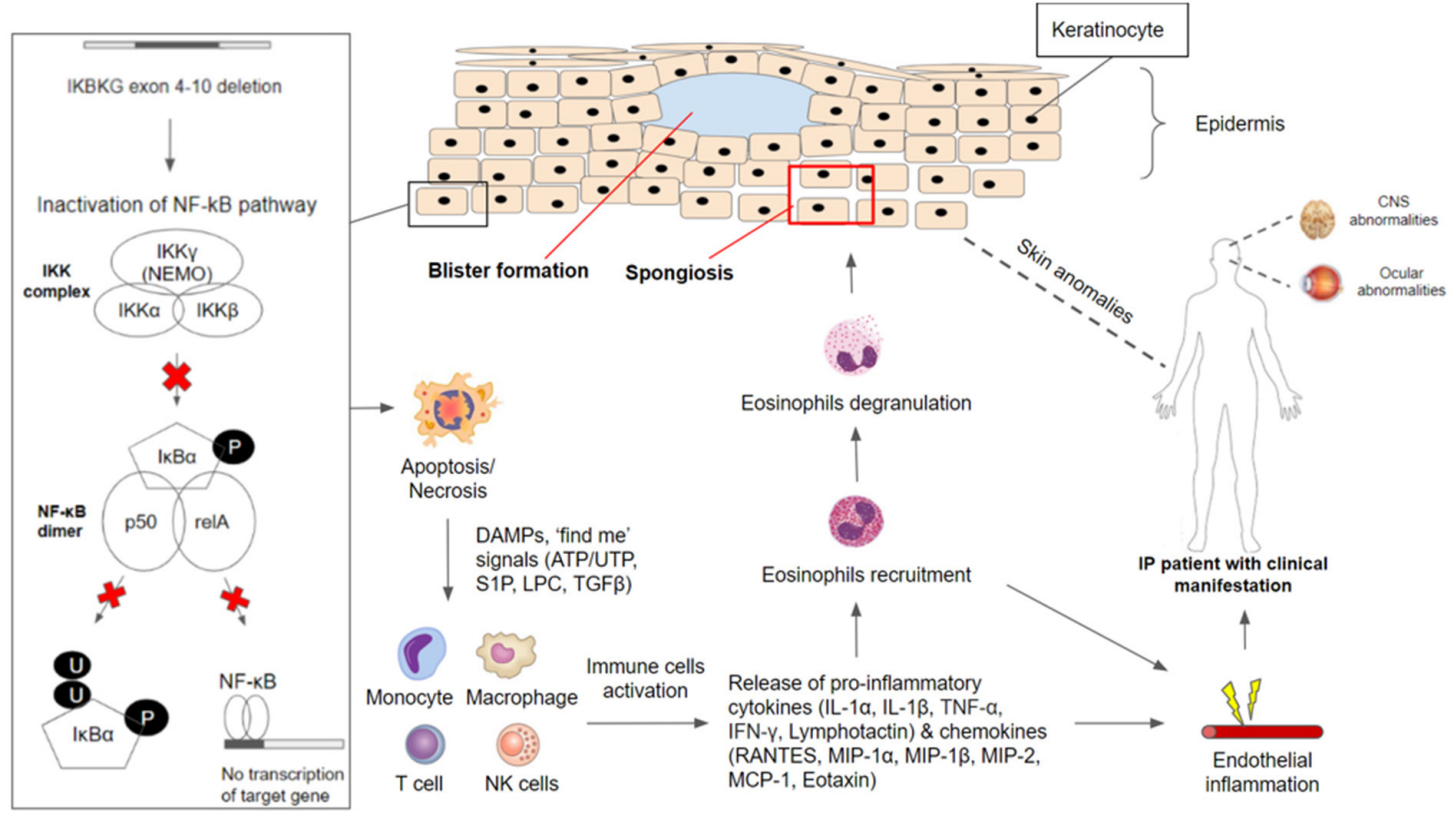
*IKBKG*/NF-κB pathophysiology in incontinentia pigmenti. The *IKBKG* gene is required for activation of the nuclear factor-kappa B (NF-κB) signaling pathway. Under non-stimulated conditions, NF-κB remained inactive in the cytoplasm through association with NEMO/IKKgamma (encoded by *IKBKG*). Phosphorylation of inhibitor NF-κB (IκB) proteins by the IKK complex results in their proteosomal degradation and subsequent release of NF-κB dimer (composed of p50 and relA subunits). Most affected individuals with IP carry a common pathogenic variant on the *IKBKG* gene with exon 4–10 deletion which caused inactivation of the NF-κB signaling pathway. *IKBKG*-deficient keratinocytes are susceptible to apoptosis/necrosis due to the loss of protection against cell death. DAMPS and ‘find me' signals (ATP/UTP, S1P, LPC, TGFβ) are released and serve as activating signals which stimulate immune-inflammatory responses. Monocytes, macrophages, T cells, and NK cells have been shown to release cytokines (IL-1α, IL-1β, TNF-α, IFN-γ, Lymphotactin) and chemokines (RANTES, MIP-1α, MIP-1β, MIP-2, MCP-1, Eotaxin), leading to the recruitment of eosinophils. Recruited eosinophils undergo degranulation to release proteases that aid in degrading adhesions between keratinocytes. This results in spongiosis and blister formation which can be observed frequently in the first stage of clinical manifestation in IP patients. Besides the major presentation of skin conditions, IP patients have often reported manifesting CNS and ocular abnormalities. NF-κB-deficient endothelial cells and other cells throughout the body have overexpression of chemotactic factors, leading to eosinophilia, which triggers extensive inflammation. Endothelial inflammation will result in vaso-occlusion and ischemia, contributing to the retinal and neurologic manifestation.

In the cases of mosaicism in males and lyonization of the X chromosome in females, the neighboring keratinocytes without *IKBKG* gene mutation expressing IKKγ (NEMO) protein can undergo NF-κB activation upon receiving activating signals from *IKBKG*-deficient keratinocytes that are undergoing apoptosis or necrosis (15). Activating signals produced from apoptotic or necrotic cells include danger-associated molecular patterns (DAMPs) as well as “find me” signals such as lysophoshatidylcholine (LCP), sphingosine 1-phosphate (S1P), nucleotide ATP/AUP and Tumor Growth Factor (TGFβ) and others (52). Activation of NF-κB in nearby *IKBKG*-expressing keratinocytes will lead to the production of chemokines such as regulated on activation, normal T cell expressed and secreted (RANTES), monocyte chemoattractant protein (MCP-1) and eotaxin which recruits eosinophils cells. Besides, pro-inflammatory cytokines such as IL-1, TNF-α, IFN-γ, Lymphotactin will be produced (50, 53–55). Studies found that IL-1 and TNF-α can upregulate eotaxin production which attracts eosinophils migration. Eosinophils recruited will undergo degranulation and the release of proteases (42, 50, 56), leading to inflammation in the epidermis and other areas of the body (Figure 1).

In the epidermis, proteases degrade tonofilaments and desmosomes which result in intracellular oedema (spongiosis) and ultimately blistering, which is observed in the first stage of IP (56, 57). Gradual clearance of skin lesions occurs upon the reduction of *IKBKG*-deficient keratinocytes due to increased apoptosis and progressive replacement by *IKBKG*-expressing keratinocytes as well as subsiding of inflammation (50, 56, 58). Moreover, TNF and other cytokines that may be produced in the epidermis during the early inflammatory phase and could play a role in the process of directly eliminating the *IKBKG*-deficient keratinocytes (58, 59). However, residual *IKBKG-*deficient keratinocytes that managed to escape and survive the elimination process can undergo second episodes of the first stage in IP due to the reoccurrence of keratinocyte hyperproliferation and subsequent inflammation reactions (50, 56).

In the event where NF-κB-deficient endothelial cells and other cells throughout the body have overexpression of chemotactic factors such as eotaxin, specific to eosinophils, this may result in systemic eosinophilia (42, 60, 61). The presence of eosinophils in combination with other inflammatory factors would lead to extensive inflammation. Endothelial inflammation will result in vaso-occlusion and ischemia, contributing to the retinal and neurologic manifestation. The occlusion of retinal arteries leads to areas of avascularity and under-perfusion, precipitating ischemia. Neovascularization occurs as sequelae to this (62). In CNS, brain atrophy and other neurological sequelae are thought to have shared similar vaso-occlusive ischemia pathophysiology in retinal ischemia events (63).

NF-κB plays a role in protecting the integrity of brain endothelial cells and the blood-brain barrier. A defect of such makes endothelial cells susceptible to a variety of potential stimuli, including infections. These stimuli upregulate proinflammatory cytokines, such as IL-6,−8, and−10, leading to endothelium inflammation and subsequent arteriopathy (64). This explains the role of systemic anti-inflammation in the treatment of neurological manifestation in IP patients. However, the exact pathogenesis in CNS lesions is still controversial.

### Genotype-phenotype correlation in incontinentia pigmenti

Studies on genotype-phenotype correlation are rare. A study on 10 Japanese patients and three of their mothers revealed no definite difference in extracutaneous manifestation between those with or without *IKBKG* gene rearrangement (30). On a separate note, a study conducted by Wang et al. (65) on 42 IP patients, identified that those with positive *IKBKG* pathogenic variants appeared to have different clinical variations in comparison to those without. It was observed that patients with positive *IKBKG* mutation had a higher frequency of hair (50 vs. 14%), dental (70 vs. 21%), ocular anomalies (45 vs. 29%), and lower frequency of CNS anomalies (20 vs. 35%) (65). This difference suggests that there is a need for in depth evaluation of the key phenotype and genotyping differences between these groups. Past studies found that the clinical phenotype of IP is widely variable as it can range from mild skin alterations (mild IP) to stroke and functional CNS abnormalities (severe IP) (25). Dangouloff and colleagues (66) reported that severe CNS abnormalities have random X-inactivation whereas no or mild CNS abnormalities have skewed inactivation. On the other hand, mutation type (common deletion vs. point mutation) was found to not correlate with disease severity (33). This may be true as the NEMO/IKKgamma protein play a role in a complex signaling pathway that regulates the expression of various genes, its mutation produces different phenotypic outcomes which may explain the entire spectrum of anomalies observed in IP (43).

A phenotype scoring system used by Fusco et al. (25) to examine the correlation between the mutation type and clinical presentation of IP patients showed a high variability of phenotype scores in patients with exon 4–10 *IKBKG* deletion and hypomorphic mutations may have broader phenotypic consequence due to it being still partially active early after the X-inactivation process. Thus, the mutations preserving some activity show an atypical phenotype characterized by the involvement of much more tissues compared to the classical IP phenotype. This can be observed in IP patients having more severe CNS and ocular defects as skewed X-inactivation is likely to modulate the severity of the disease (25). Besides, the variability in disease expression for patients carrying the same *IKBKG* mutation in different genomic backgrounds may be explained by the additional genetic factors such as modifier genes observed in many mendelian diseases (53). To date, there is no significant genotype-phenotype relation in IP. However, studies have proposed that a combination of the mutation type, the function domain affected, X-inactivation and genomic background may lead to the variability observed in IP phenotypes (25, 33, 53). Comparisons are detailed in Table 1.

**Table 1:** Extracutaneous difference between common exon 4-10 deletion vs. others.

**(*n*), population, gender**	**Results (positive result/total, %)**	**Extracutaneous phenotype**, ***n*** **(%)**		**Conclusion**	**References**
		**Positive genetic test (delExon 4–10)**	**Negative genetic test (Exon 4–10)**		
*n* = 10 Japanese, F	5/10, 50%	Alopecia, 3/5 (60) Opthalmic manifestation 3/5 (60) Neurological manifestation 1/5 (20) CVS manifestation—none No dental and nail abnormalities described	Alopecia—none Ophthalmic manifestation 1/5 Neurological manifestation 1/5 CVS manifestation—none	No definite difference in extracutaneous manifestation in their studies and others	(30)
*n* = 42, M & F White, 27/42 (64%) Hispanic, 1/42 (2%) Asian, 9/42 (21%) Black, 4/42 (10%)	20/34, 58.8%	Hair−10/20 (50) Nail−2/20 (10) Dental-−14/20 (70) Palate−0/20 (0) Ocular−9/20 (45) CNS−4/20 (20)	Hair−2/14 (14) Nail−2/14 (14) Dental−3/14 (21) Palate−1/14 (7) Ocular−4/14 (29) CNS−5/14 (36)	There is a clinical difference between *IKBKG* pathogenic variant positive and negative IP cohort	(65)
*n* = 25, Korean, F	20/25, 80% (common Exon 4–10) 5/25, 20% (intragenic sequence variants)	Hair−5/20 Nail−2/20 Dental−3/20 Ocular−4/20 CNS−5/20	Hair−0/5 Nail−0/5 Dental−0/5 Ocular−3/5 CNS−2/5	No statistically significant differences in frequencies of extracutaneous manifestations or phenotype scores	(33)
*n* = 122, France, Detailed phenotype described only in 60 patients	73/122, 59.8%	Hair−8/50 (16) Nail−7/50 (14) Dental−22/50 (44) Ocular−8/50 (16) CNS−4/50 (8)	Only described those with novel mutation identified (*n* = 10/49, 40.2%)—small nucleotide mutation, K90 (266-269delAGA) and H360MfsX449 (1077–1078delC)—high phenotype score compared to those with missense mutation (169G → A, 367 → T)) or non-sense mutation (715C → T, 1150 C → T) Nervous system defects were observed in 44% of patients carrying point-mutations and ocular defects in 55% of patients, whereas only 8% of IP patients with genomic deletion suffered nervous system defects and 16% had ocular defects.	Patients with in frame deletion mutation K90 (266-269delAGA) and frameshift mutations such as H360MfsX449 (1077-1078delC) & P372PfsX450 (1115-1116delT) suffer more severe disease compared with patients with missense or non-sense mutations	(25)
*n* = 18, France, F	All	Only included neurological symptoms		Mutation type (delete vs. missense/non-sense) had no correlation with MRI Random X inactivation had more severe MRI anomalies	(66)
	15/18, 83% (common Exon 4–10) 3/18, 17% (missense or non-sense mutation)	3 main neuroimaging identified Normal (5/18) Mild anomalies of periventricular white matter with T2 weighted hyper signal (7/18), mild cortical atrophy (5/7), and atrophy of corpus callosum (5/7) Severe cortical anomalies suggestive of vascular diseases (7/18)			

M, Male; F, Female; CNS, Central Nervous system.

## Current treatment strategies

The current treatment strategies require multidisciplinary experts (including but not limited to dermatology, neurology, pediatric, geneticist, and ophthalmologist). The treatment approach involves symptom control, rehabilitation, and preventing complications (1).

Among those with skin presentation severely inflamed verrucous lesions can be treated with topical or systemic steroids and/or topical calcineurin inhibitors. Retinoids have been reported to regress painful, verrucous tumors. Physicians shall not be tempted to treat pigmentation with lasers, it may potentially flare skin inflammation. Photoprotection should be emphasized as ultraviolet exposure was found to aggravate cutaneous lesions (1).

An eye examination should be done as soon as IP diagnosis is concluded as this may be visual protective. A protocol used to screen for retinopathy of prematurity should be utilized. Evidence of peripheral vasculopathy warrants an examination under general anesthesia with fundus photography and fluorescein angiography. Argon laser can be used to treat the non-perfusion zone and repeated laser photocoagulation may be required. Ranibizumab had been described to treat refractory proliferative retinopathy (67) adjunctive to failed laser photocoagulations. Strabismus and retinal detachment can be repaired through surgery (68, 69). Propranolol was mentioned as a potential treatment for retinopathy of prematurity (70).

Early neonatal neurological manifestation determines long term patient prognosis and occurrence of disabilities. Most that without neonatal CNS abnormalities usually have normal physical and cognitive development. Thus, it is crucial for a detailed early neurological examination to be done after an accurate dermatological examination. Seizures should be investigated with an electroencephalogram (EEG) and a brain MRI. The two main treatment objectives during the neonatal period include antiepileptic treatment and anti-inflammatory drugs. Antiepileptic of choice will depend on the seizure semiology and the age of the patient (71–73), while steroid is the anti-inflammatory drug of choice. Recently, anti-TNF had been used with success. Gene therapy is under investigation for its potential in correcting severe cerebrovascular pathology (74, 75). Those who suffer from neurological sequelae should be managed by a rehabilitation team including a physician, physiotherapist, speech therapist, and occupational therapist as early as possible to alleviate neurocognitive and orthopedic complications. Those without neurological manifestation should still be routinely followed up in order to detect new neurological, neurocognitive, and/or epileptological manifestations.

Children should be under have a regular dental follow-up to pick up dental manifestation and maintain teeth functioning. Issues that may arise include multiple agenesis, coronary morphological abnormalities, dentofacial orthopedics anomalies, and delayed or absent tooth eruption. Interim dentures and prosthodontic treatment could be used to replace lost dentition and for tooth relocation and alignment. Definitive implant-prothetic and orthodontic rehabilitation can be initiated when growth has halted. Multidisciplinary assessment involving an implantologist periodontologist, and specialist in dentofacial orthopedics and prosthesis may be required (1).

## Future directions and conclusion

Aside from having PCR and Sanger sequencing as the gold standard method for genetic testing in IP, further innovation and advancement of NGS with established strategies are needed to increase the sensitivity and specificity of IP molecular diagnosis. Clinical variations between positive and negative *IKBKG* pathogenic variant cohorts indicate the need for in-depth analysis of the key genotypic and phenotypic differences between these groups. A greater extent of understanding of the genotype-phenotype correlation of IP will support clinicians to direct investigations and counseling for affected individuals and their families regarding prognosis and future reproductive choices. Lastly, clinical treatment which involves identifying possible early immunosuppressants to reduce inflammatory markers could be a potential treatment strategy to reduce disability leading, such as retinal and cerebral ischemia. This may aid in the prevention and optimal management of serious complications of IP.

## Author contributions

WY and KH conceptualized the project. KH, HL, ZP, and WY wrote the manuscript. HL designed the figure and prepared the table. KH, WY, HL, ZP, KL, and ZL provided vital guidance and insight to the work. All authors contributed to manuscript revision, read, and approved the submitted version.

## Funding

This work was supported by the Ministry of Education (MOE) under the Fundamental Research Grant Scheme (FRGS/1/2019/SKK08/TAYLOR/02/2) awarded to WY, Fundamental Research Grant Scheme (FRGS/1/2020/SKK01/UPM/02/1) awarded to KH, and Fundamental Research Grant Scheme (FRGS/1/2019/STG05/TAYLOR/03/3) awarded to ZL.

## Conflict of interest

The authors declare that the research was conducted in the absence of any commercial or financial relationships that could be construed as a potential conflict of interest.

## Publisher's note

All claims expressed in this article are solely those of the authors and do not necessarily represent those of their affiliated organizations, or those of the publisher, the editors and the reviewers. Any product that may be evaluated in this article, or claim that may be made by its manufacturer, is not guaranteed or endorsed by the publisher.
