# Supplementary material for: Uncovering incontinentia pigmenti: From DNA sequence to pathophysiology
Source: Front Pediatr. 2022 Sep 6;10:900606. doi: 10.3389/fped.2022.900606 (PMC9485571; doi:10.3389/fped.2022.900606)
Supplement: Supplementary file 1 [file Table_1.DOCX]

Supplementary Table 1: Types of *IKBKG* gene mutation in Incontinentia Pigmenti patients from different population

| **Associated Population** | **Total number of IP cases [n]** | | **Number of cases with the mutation involved [%]** | **Exon/Intron Involved** | **Type of Mutation Involved** | **References** |
| --- | --- | --- | --- | --- | --- | --- |
| European | | [n = 122] | 73 (59.8%) | Exon 4 - 10 | Deletion | 14 |
|  |  | [n = 193] | 145 (75.1%) | Exon 4 – 10 | Deletion | 15 |
|  |  | [n = 7] | 7 (100%) | N/A | Deletion | 18 |
|  |  | [n = 7] | 1 (14.3%) | Exon 1B – 1C | Deletion | 18 |
| Chinese | | [n = 21] | 13 (61.9%) | Exon 4 – 10 | Deletion | 22 |
|  |  | [n = 12] | 3 (25%) | Exon 4 – 10 | Deletion | 44 |
|  |  | [n = 21] | 1 (4.7%) | Exon 5 | Missense Mutation (Q183H) | 22 |
|  |  | [n = 21] | 1 (4.7%) | Exon 5 | Point Mutation (G539C) | 22 |
|  |  | [n = 12] | 1 (8.3%) | Intron 8 | Single Nucleotide Polymorphism  (21690 T to C mutation) | 44 |
|  |  | [n = 12] | 1 (8.3%) | Exon 6 | Single Nucleotide Mutation (deletion of 19545 T) | 44 |
| Japanese | | [n = 30] | 13 (43.3%) | Exon 4 – 10 | Deletion | 20 |
|  |  | [n = 10] | 5 (50%) | Exon 4 - 10 | Deletion | 29 |
|  |  | [n = 5] | 4 (80%) | Exon 4 - 10 | Deletion | 47 |
|  |  | [n = 30] | 3 (10%) | Exon 4 - 10 | Nonsense Mutation (c.343A>T, c.268A>T, c.184C>T) | 20 |
|  |  | [n = 30] | 1 (3.3%) | Exon 4 – 10 | Frameshift Mutation (c.896delC) | 20 |
|  |  | [n = 30] | 1 (3.3%) | Exon 4 - 10 | In-frame amino acid deletion (c.976_978delAAG) | 20 |
|  |  | [n = 30] | 1 (3.3%) | Exon 4 - 10 | Splice mutation (c.913‐2A>G) | 20 |
| Korean | | [n = 4] | 4 (100%) | Exon 4 - 10 | Deletion | 48 |
|  | | [n = 25] | 20 (80%) | Exon 4 – 10 | Deletion | 61 |
|  | | [n = 25] | 1 (4%) | Exon 4 | Missense mutation (c.518G>A) | 61 |
|  | | [n = 25] | 1 (4%) | Exon 2 | Nonsense mutation (c.184C>T) | 61 |
|  | | [n = 25] | 2 (8%) | Exon 2, Exon 7 | Frameshift mutation (c.151dupC, c.840delG) | 61 |
|  | | [n = 25] | 1 (4%) | Exon 2 | Nonsense mutation (c.154C>T) | 61 |
| Indian | | [n = 4] | 4 (100%) | Exon 4 – 10 | Deletion | 40 |
